# The impact of long-lasting microbial larvicides in reducing malaria transmission and clinical malaria incidence: study protocol for a cluster randomized controlled trial

**DOI:** 10.1186/s13063-016-1545-4

**Published:** 2016-08-25

**Authors:** Guofa Zhou, Virginia Wiseman, Harrysone E. Atieli, Ming-Chieh Lee, Andrew K. Githeko, Guiyun Yan

**Affiliations:** 1 Program in Public Health, University of California, Irvine, USA; 2 School of Public Health & Community Medicine, University of New South Wales, Sydney, Australia; 3 Department of Global Health and Development, London School of Hygiene & Tropical Medicine, London, UK; 4 Centre for Global Health Research, Kenya Medical Research Institute, Kisumu, Kenya; 5 Maseno University, Kisumu, Kenya

**Keywords:** Long-lasting microbial larvicide, Cluster-randomized controlled trial, Vector abundance, Malaria transmission intensity, Clinical malaria, Cost-effectiveness

## Abstract

**Background:**

The massive scale-up of insecticide-treated nets (ITNs) and indoor residual spraying (IRS) has led to a substantial increase in malaria vector insecticide resistance as well as in increased outdoor transmission, both of which hamper the effectiveness and efficiency of ITN and IRS. Long-lasting microbial larvicide can be a cost-effective new supplemental intervention tool for malaria control.

**Methods/design:**

We will implement the long-lasting microbial larvicide intervention in 28 clusters in two counties in western Kenya. We will test FourStar controlled release larvicide (6 % by weight *Bacillus thuringiensis israelensis* and 1 % *Bacillus sphaerius*) by applying FourStar controlled release granule formulation, 90-day briquettes, and 180-day briquettes in different habitat types. The primary endpoint is clinical malaria incidence rate and the secondary endpoint is malaria vector abundance and transmission intensity. The intervention will be conducted as a two-step approach. First, we will conduct a four-cluster trial (two clusters per county, with one of the two clusters randomly assigned to the intervention arm) to optimize the larvicide application scheme. Second, we will conduct an open-label, cluster-randomized trial to evaluate the effectiveness and cost-effectiveness of the larvicide. Fourteen clusters in each county will be assigned to intervention (treatment) or no intervention (control) by a block randomization on the basis of clinical malaria incidence, vector density, and human population size per site. We will treat each treatment cluster with larvicide for three rounds at 4-month intervals, followed by no treatment for the following 8 months. Next, we will switch the control and treatment sites. The former control sites will receive three rounds of larvicide treatment at appropriate time intervals, and former treatment sites will receive no larvicide. We will monitor indoor and outdoor vector abundance using CO_2_-baited CDC light traps equipped with collection bottle rotators. Clinical malaria data will be aggregated from government-run malaria treatment centers.

**Discussion:**

Since current first-line vector intervention methods do not target outdoor transmission and will select for higher insecticide resistance, new methods beyond bed nets and IRS should be considered. Long-lasting microbial larviciding represents a promising new tool that can target both indoor and outdoor transmission and alleviate the problem of pyrethroid resistance. It also has the potential to diminish costs by reducing larvicide reapplications. If successful, it could revolutionize malaria vector control in Africa, just as long-lasting bed nets have done.

**Trial registration:**

U.S. National Institute of Health, study ID NCT02392832. Registered on 3 February 2015.

## Background

In the past decade, the massive scale-up of insecticide-treated bed nets (ITNs) and indoor residual spraying (IRS), together with the use of artemisinin-based combination treatments, have led to major changes in malaria epidemiology and vector biology. Overall malaria prevalence and incidence have been greatly reduced worldwide [1]. But the reductions in malaria have not been achieved uniformly; some sites have experienced continued reductions in both clinical malaria and overall parasite prevalence [2–6], while other sites showed stability or resurgence in malaria despite high coverage of ITNs and IRS [7–12]. Persistence and resurgence of vector populations continues to be an important issue for malaria control and elimination [12–16]. More importantly, extensive use of ITNs and IRS has created intensive selection pressures for malaria vector insecticide resistance as well as for potential outdoor transmission, which appears to be limiting the success of ITNs and IRS. For example, in Africa, where malaria is most prevalent and pyrethroid-impregnated ITNs have been used for more than a decade, there is ample evidence of the emergence and spread of pyrethroid resistance in *Anopheles gambiae* s.s., the major African malaria vector, as well as in *An. arabiensis* and *An. funestus* s.l. [17–20]. Both the prevalence of *An. gambiae* s.s. resistance to pyrethroids and DDT and the frequency of knock-down resistance (*kdr*) have reached alarming levels throughout Africa from 2010–2012 [18]. Unfortunately, pyrethroids are the only class of insecticides that the World Health Organization (WHO) recommends for the treatment of ITNs [21]. Furthermore, a number of recent studies have documented a shift in the biting behavior of *An. gambiae* s.s. and *An. funestus*, from biting exclusively indoors at night to biting both indoors and outdoors during early evening and morning hours when people are active but not protected by IRS or ITNs, or to biting indoors but resting outdoors [22–24]. Apart from these intraspecific changes in biting behavior, shifts in vector species composition, i.e., from the previously predominant indoor-biting *An. gambiae* s.s. to the concurrently predominant species *An. arabiensis*, which prefers to bite and rest outdoors in some parts of Africa, can also increase outdoor transmission [25–28]. Because IRS and ITNs have little impact on outdoor-resting and outdoor- and early-biting vectors, outdoor transmission represents one of the most important challenges in malaria control. New interventions are urgently needed to augment current public health measures and reduce outdoor transmission [29].

Larval control has historically been very successful and is widely used for mosquito control in many parts of the developed world [30–33], but is not commonly used in Africa. Field evaluation of anopheline mosquitoes in Africa found that larviciding was effective in killing anopheline larvae and reducing adult malaria vector abundance in various sites [34–39]. Microbial larvicides are effective in controlling malaria vectors, and they can be used on a large scale in combination with ongoing ITN and IRS programs [35, 38, 40]. However, conventional larvicide formulations are associated with high material and operational costs due to the need for frequent habitat re-treatment, i.e., weekly re-treatment, as well as logistical issues in the field [34–36, 40, 41]. Recently, an improved slow-release larvicide formulation was field-tested for controlling *Anopheles* mosquitoes, yielding an effective duration of approximately 4 weeks [42]. Considering the monthly reapplication interval, this still may not be a cost-effective product for large-scale application. The new US EPA-approved long-lasting formulation, FourStar Microbial Briquets (Central Life Sciences, Sag Harbor, NY, USA), is potentially effective for up to 6 months (http://www.centralmosquitocontrol.com/all-products/fourstar/fourstar-briquet-180), and preliminary data suggest that it is effective in malaria mosquito control [GZ, unpublished data]. Field-testing is needed to determine the efficacy and cost-effectiveness of this long-lasting larvicide.

The central objective of this study is to determine the effectiveness and cost-effectiveness of long-lasting microbial larviciding (LLML) on the incidence of clinical malaria and the reduction of transmission intensity. The hypothesis is that adding LLML to ongoing ITN and IRS programs will lead to significant reductions in both indoor and outdoor malaria transmission and malaria incidence as well as cost savings. This paper describes a protocol for evaluating the impact of LLML in reducing malaria vector populations and clinical malaria incidence.

## Methods/design

### Hypothesis, larvicides, and endpoint outcomes

#### Hypothesis

The addition of LLML to ongoing ITN and IRS programs will lead to significant reductions in both indoor and outdoor malaria transmission and malaria incidence.

#### Objective

The central objective of this trial is to determine the impact and cost-effectiveness of LLML in reducing malaria transmission and clinical malaria incidence in Africa.

#### Trial design

This is an open-label, cluster-randomized controlled trial with two arms and a baseline period which allows for crossover.

#### Microbial larvicide formulations

We will test the FourStar LLML manufactured by Central Life Sciences. The active ingredients are *Bacillus thuringiensis israelensis* (*Bti*) (6 % by weight) and *Bacillus sphaerius* (*Bs*) (1 % by weight). We will treat temporary, semipermanent, and permanent habitats with controlled-release granule formulation, 90-day briquettes, and 180-day briquets, respectively. Application dosage will follow the recommendation of the manufacturer: 10 lbs per acre of water surface for the granule formulation, and one briquette per 100 ft^2^ of water surface for the briquette formulations, regardless of water depth. Re-treatment will occur at a frequency of 4 to 5 months.

#### Primary and secondary endpoint

The *primary endpoint* is the clinical malaria incidence rate. The *secondary endpoint* is the malaria vector abundance and transmission intensity. A clinical malaria case is defined as an individual with fever (axillary temperature of 37.5 °C or higher) and other related symptoms such as chills, severe malaise, headache, or vomiting in the presence of a *Plasmodium*-positive blood smear. The clinical malaria incidence rate is calculated as the number of clinical malaria episodes divided by the total person time (person years) at risk based on demographic surveys. Malaria vector abundance is measured as the total density of malaria vector mosquitoes (*An. gambiae* s.s., *An. arabiensis*, *An. funestus*, and other new species capable of transmitting malaria) collected indoors and outdoors by CO_2_-baited light traps. Malaria transmission intensity is measured as the sum of the indoor and outdoor entomological inoculation rates (EIRs).

### Study area

**Fig. 1:**
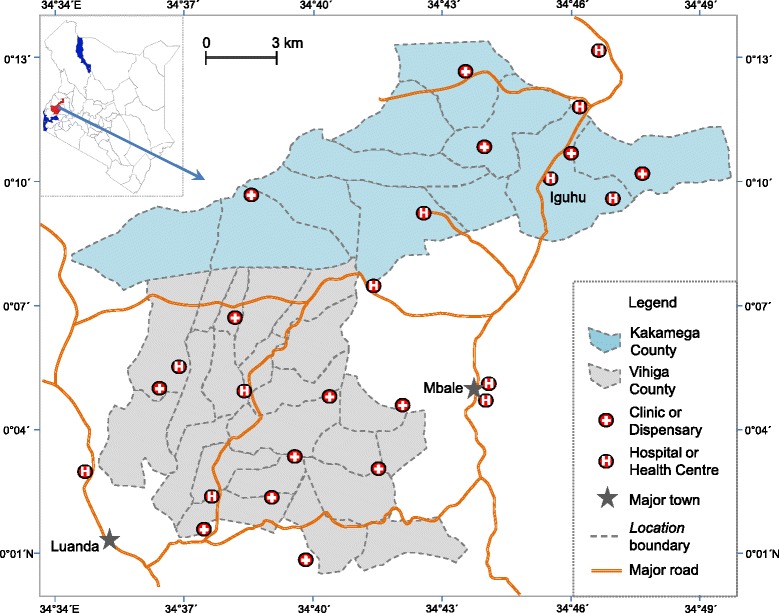
Map of study area and distribution of clinics and hospitals with catchment areas overlapping the study area

We will conduct our study in 28 randomly selected clusters in the highland localities (1400 m to 1600 m altitude) of Kakamega and Vihiga counties, western Kenya (34°34’ to 34°50’E, 0°00’ to 0°12’N) (Fig. 1). A cluster typically consists of an area of approximately 4 km^2^ in size and comprises 400–700 households and about 2000–3000 residents. The catchment population of the study area, including intervention clusters, control clusters, and buffer zones, is estimated as 250,000 according to 2010 census data.

Local residents are predominantly farmers and depend upon farming, cattle and goat herding for subsistence. Malaria transmission is seasonal, with two peaks in vector abundance reflecting the bimodal rainfall pattern: a major peak between April and June and a minor peak between October and November [43]. Most malaria is caused by *Plasmodium falciparum* [12]. The main malaria vectors in the area are *An. gambiae* s.s., *An. arabiensis*, and *An. funestus* s.l. [43, 44]. Malaria vector density was high in the early 2000s, decreased substantially during 2006–2008 after the first round of mass distribution of ITNs in 2006, and has gradually increased since 2008 [12]. Pyrethrum spray collections (PSC) of indoor-resting *Anopheles* were about 1.0 females/house/night in 2014 compared to 0.1 females/house/night in 2007 [12, 45]. Cross-sectional community-level surveys in May 2011 indicated that parasite prevalence averaged 11.8 % in the general population (all ages) but varied between localities from 3.3 % to 25.4 % [44]. In school children aged 6–13 years, surveys in 2012 found an average parasite prevalence of 27.2 %, which varied from 18.8 to 35.4 % among villages [45, 46]. Active case surveillance through bi-weekly home visits in May 2011 indicated an average annual clinical malaria incidence rate of 31.4 cases per 1000 people in the general population, varying from 28.9 to 36.2 between villages [47]. Ownership of ITNs (mainly long-lasting insecticidal nets) ranged from 78.3 to 84.2 % in 2013 [48]. There have been several attempts in the past 10 years to control malaria vectors in the study area using conventional formulations of *Bti*/*Bs* (i.e., through weekly re-treatment of larval habitats) and IRS [35, 39, 44, 49]. The last community-wide mass distribution of ITNs was undertaken by the Division of Malaria Control (DOMC) of Kenya in 2014. Currently there is no mass distribution of ITNs or IRS and no larviciding in the proposed study area.

### Demographic survey and cluster definition

For purposes of planning and conducting an evaluation of the intervention, we will subdivide the field area into villages (clusters), which is the smallest administrative unit in Kenya. Using villages as clusters has advantages over random sampling. First, the clinical records in health centers or hospitals in Kenya generally include the name of the village and sublocation (the next-highest administrative level); therefore, clinical malaria cases can be traced back to the village level. Second, villages have been conveniently used as intervention/control clusters in previous trials [15, 50].

**Fig. 2:**
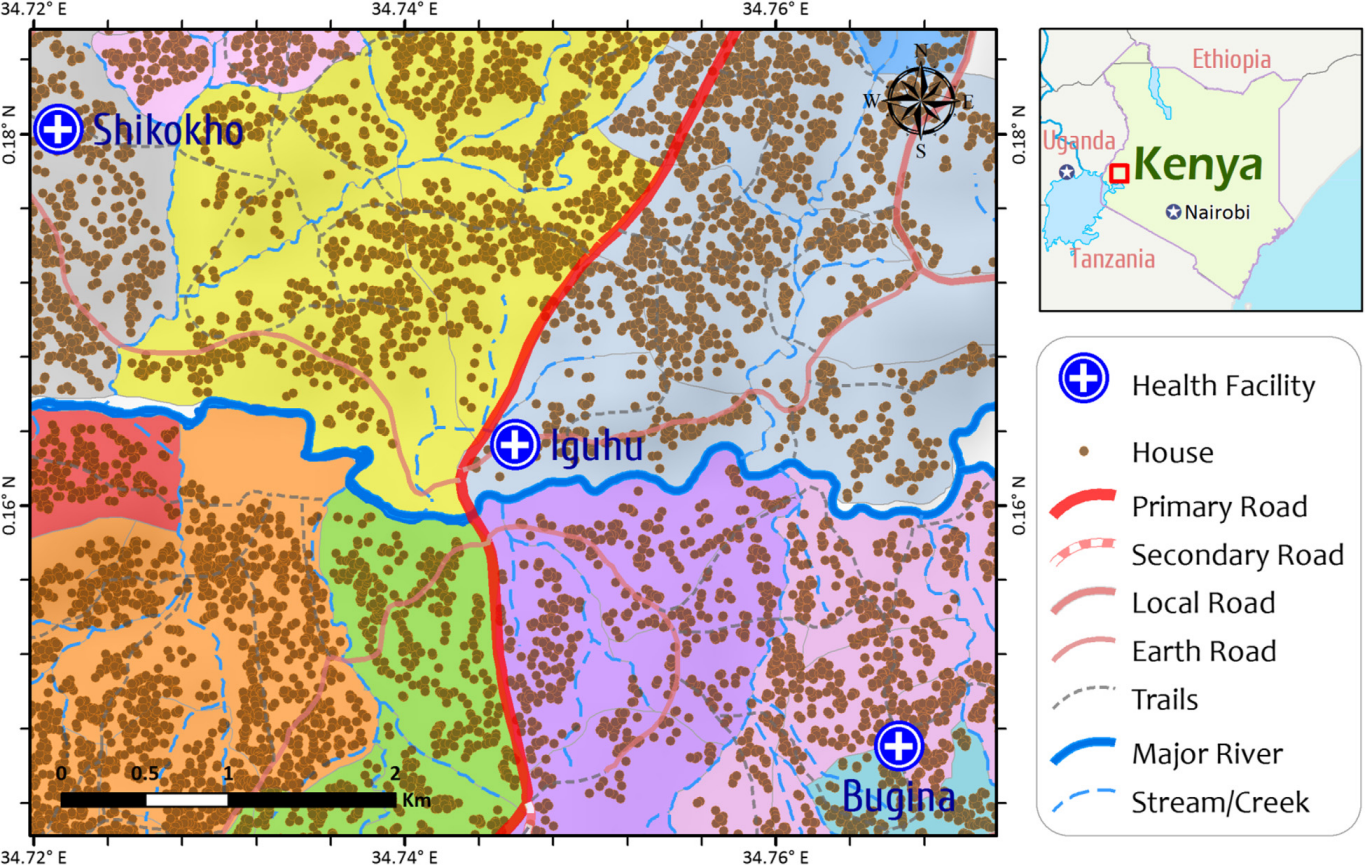
A section of study area showing the distribution of households and clusters for field evaluation of long-lasting microbial larvicides. The area with a unique color represents a cluster

**Fig. 3:**
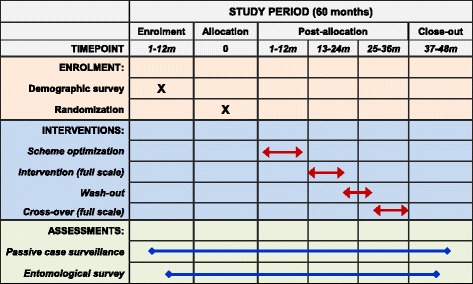
Timeline for the schedules of enrollment, interventions, and assessments

Our field team will conduct the demographic surveys before the start of the intervention. Each team will be provided with a printed overview map (Figs. 1 and 2) and a handheld Google Nexus 7 tablet. A surveillance team, comprising a field technician, a reporter, and a local guide, will visit every compound to explain the study procedures, tally inhabitants, and collect information on house characteristics. If the head of the compound agrees to participate, we will record the geographical coordinates of the main house of the compound and compound codes will be written in permanent marker on the front wall next to the door. We will record the genders and ages of all compound members on questionnaire forms using the on-site Google Nexus 7, which will update the database in real time together with the GPS coordinates of the surveyed compound. We will map the locations of all compounds using ArcGIS 10 (Fig. 2). Demographic surveillance will be done in year 1, 6–12 months prior to intervention (Fig. 3).

We will draw village boundaries based on the demographic surveys and confirm it with the field teams and the database manager. If a village is too small (i.e., fewer than 100 households, or fewer than 500 inhabitants, or with an area less than 1 km^2^), we will combine the village with a neighboring village to form one cluster. Total and age- and gender-specific populations will be aggregated at the cluster level.

### Clinical malaria records collection

Clinical malaria records will be collected from 8 to 12 months prior to intervention, to calculate baseline incidence rate at each cluster for cluster randomization, through to 8 to 12 months after all interventions (Fig. 3). We will collect information on clinical malaria cases retrospectively from all government-run hospitals, health care centers, and clinics located either within the study area itself or within catchment areas overlapping the study area. We will obtain clinical data from the treatment centers through the malaria control office of Kakamega and Vihiga counties, Kenya. We will also collect patient- and treatment-related information, including age, gender, date of diagnosis, parasite species, village of patient (or sublocation if village is missing), and prescriptions given. All personal identifiers will be excluded from this study. A clinical malaria case is defined as an individual with fever (axillary temperature of 37.5 °C or higher) and other related symptoms such as chills, severe malaise, headache, or vomiting in the presence of a *Plasmodium*-positive blood smear [47]. The clinical malaria incidence rate is calculated as the number of clinical malaria episodes divided by the total person time (person years) at risk based on demographic surveys [47]. We will also collect the aggregated monthly diarrhea data at each site along with clinical malaria records from local health clinics and hospitals. We will not conduct prospective passive surveillance, active home visits, or cross-sectional blood surveys.

We will calculate the clinical malaria incidence rate separately for each cluster, different study period and different age group (i.e., under 5, 5–14, over 14 years). We will include all clinical malaria cases in our study, including cases diagnosed during the four study periods (Fig. 3): (A) preintervention period: baseline clinical malaria records started at least 8–12 months prior to the application of long-lasting microbial larvicides till intervention, (B) intervention period: all clinical records during the intervention period, (C) the 8-month wash-out period, and (D) postintervention period: clinical malaria records till 8–12 months after the last round of larvicide application.

### Ethical and conflict of interest statement

Permission to use microbial larvicides for malaria vector control has been obtained from the Pest Control Products Board of Kenya. Ethical clearance has been approved by the Scientific and Ethical Unit of the Kenya Medical Research Institute (KEMRI). As described, aggregated clinical data will be obtained from the treatment centers through the malaria control offices of Kakamega and Vihiga counties, Kenya. According to US Department of Health and Human Services Code of Federal Regulations 45 CFR 46.101(b) part 4 (Categories of Exempt Human Subjects Research), these data are in the category of exempt human subjects research, which involves the study of existing data, documents, or records, with no collection of subject-level information. Informed consent will be obtained from each participant. All investigative team members in the United States, Kenya, and Australia have no financial conflict of interest with the larvicide manufacturer, Central Life Sciences.

### Malaria vector population monitoring

We will conduct baseline malaria vector surveillance at least 4 months prior to any application of LLMLs (Fig. 3). We will conduct malaria vector population surveillance on a monthly basis continuously till at least 8 months after the last round of larvicide application (Fig. 3). We will monitor both indoor- and outdoor-biting mosquito abundance using CO_2_-baited Centers for Disease Control (CDC) light traps equipped with collection bottle rotators (Model 1512, John W. Hock Co., Gainesville, FL, USA). The collection bottle rotator, which has eight separate plastic collection bottles, will be programmed to collect active mosquitoes at 2-h intervals between 16:00–08:00. We will place two traps within each sampling compound: one inside the living room, the other outside the house 5 m away. We will conduct a total of 64 trap-nights of vector sampling per cluster per month. This will provide an estimation precision of 0.2 mosquitoes using the previously determined standard deviation [51]. Species of collected mosquitoes will be identified and blood-feeding status will be recorded. We will test for *P. falciparum* sporozoite infection and blood meal source using an enzyme-linked immunosorbent assay (ELISA) on all specimens [52, 53]. For each house where the vector population was sampled, we will record the number of sleeping persons at each house on the same day as the vector survey. We will calculate sporozoite rate and EIR for each cluster. EIRs will be calculated as (the number of *Anopheles* per person) × (the average number of persons bitten by one *Anopheles* in 1 day) × (sporozoite rate), and standardized to a monthly basis. The trapping method will allow for comparison of indoor- and outdoor-biting mosquito abundance and determination of nightly biting activity patterns. We will calculate indoor and outdoor transmission intensities separately assuming that all mosquitoes collected from a compound had their blood meal from the same household. We will calculate EIR for the four study periods as describe above: (A) preintervention period: baseline vector surveillance started at least 6 months prior to the application of long-lasting microbial larvicides till intervention, (B) intervention period, (C) the 8-month wash-out period, and (D) postintervention period: vector surveillance continued till 8 months after the last round of larvicide application.

To determine whether new malaria vector species are present in the study sites, we will sequence the ribosomal second internal transcribed spacer (ITS2) and mitochondrial *CO1* gene in anopheline specimens that are not amplified by the recombinant deoxyribonucleic acid polymerase chain reaction (rDNA-PCR) method, and we will conduct phylogenetic analysis to determine whether the new species found by Stevenson et al. are also present in the study sites [54].

### Intervention design

We will conduct the intervention using a two-step approach. First, we will conduct a small-scale four-cluster trial to optimize the time, duration, and quantity of LLML application. Second, we will conduct a cluster-randomized trial to test the effectiveness and cost-effectiveness of LLML. The design has two parallel arms, i.e., control and intervention, and allows for baseline survey without intervention and crossover (Fig. 3).

#### Small-scale entomological evaluation

We will select four clusters, two in each county, for an entomological evaluation of the optimal larvicide application scheme (Fig. 3). We will randomly select two clusters, one in each county, treated with larvicides (intervention) and the other two sites will serve as controls (no intervention). We will treat temporary habitats with FourStar controlled release granule formulation, which maintains effectiveness through wet and dry periods for up to 1 month. We will treat semipermanent habitats with FourStar 90-day briquettes and permanent habitats with FourStar 180-day briquettes. Application dosage will follow the recommendation of the manufacturer, Central Life Sciences: 10 lbs per acre of water surface for the granule formulation, and one briquette per 100 ft^2^ of water surface for the briquette formulations, regardless of water depth. We will re-treat the habitats every 4 to 5 months. On a weekly basis in the treatment and control sites, we will use aerial samplers to determine habitat pupal productivity, and use standard dippers to determine larval abundance. This will allow for determination of habitat productivity with a tolerable error of 0.5 mosquitoes, based on the standard deviation identified in previous studies [55]. We will monitor indoor and outdoor vector abundance using 64 trap-nights per cluster per month. This sample size will allow detection of a difference in average vector abundance of 0.12 mosquitoes with 80 % statistical power and 0.05 type-I error. We will use ELISA methods to determine *Anopheles* mosquitoes’ sporozoite infection and blood-feeding host preference [43].

We will analyze the data immediately after the small-scale trial (Fig. 3) using analysis of variance (ANOVA) with repeated measures and appropriate transformation to determine the effects of habitat larviciding on mosquito abundance and transmission intensity [56]. The percentage reduction in malaria transmission intensity will be calculated [57].

#### Randomization

We will assign fourteen clusters each in the two counties to intervention (treatment) or no intervention (control) by a block randomization method on the basis of clinical malaria incidence, vector density, and human population size per site. Year 1 will focus on preparing the study sites and working with clinics and hospitals to help them improve their routine malaria surveillance (Fig. 3). In year 2, we will conduct preliminary surveys on all 28 sites to determine (1) clinical malaria incidence, (2) vector density, (3) geographic information system (GIS) coordinates of larval habitats, and (4) human population size. Human population size for each cluster, stratified into three age groups (under 5 years, 5–15 years and over 15 years) will be ascertained from our existing data. We will obtain age-group level aggregated morbidity data (the number of clinical malaria cases per age group, without identifiers) from local hospitals and clinics where the sampled residents seek treatment. These clinical data are reported to the Ministry of Health of Kenya and hence are publicly available. We will determine vector abundance using CO_2_-baited CDC light traps for 16 trap-nights per cluster per month in each of the indoor and outdoor environments.

Using these data, each cluster will be allocated to either treatment or control through randomization using the following procedures. First, each of the four parameters listed above will be standardized with the highest cluster as 1. Second, we will assign the highest weight for clinical malaria cases (weight = 5), the lowest weight for human population size (0.5), and intermediate weights for expected vector density (2) and larval habitats (2), following the method of Corbel et al*.* [58]. For each cluster a rank score will be computed as the sum of weighted clinical malaria incidence, vector density, habitat abundance, and human population size. Finally, the 14 clusters within each county will be sequentially numbered according to their rank scores and sorted into seven blocks of two clusters having successive rank scores. We expect the two clusters within each block to have similar risk characteristics for clinical malaria, vector abundance, larval habitats, and human population size. In each block, the ranks of the two clusters are put into two sealed envelopes, one cluster will be randomly allocated to treatment and another to control, using computer-generated random numbers (0 – control, 1 – intervention).

#### Intervention strategy and regime

After the larvicide application optimization and study cluster randomization, we will treat each treatment cluster with LLML at the time interval of 4 or 5 months (Fig. 3). The first treatment will be conducted in February-March about 1 month before the beginning of the long rainy season which usually starts in April. After three treatments, we will perform no treatments for the next 8 months. This will provide useful data on the dynamics of action of the LLML and the waning efficacy of LLML over time. These data will be important in analyzing cost-effectiveness to help optimize the timing of re-treatments. After 8 months, a total washout of the LLMLs will be assumed to have taken place. Next, we will perform a crossover and switch of the control and treatment clusters. Former control clusters will receive three rounds of LLML treatment at appropriate time intervals, and the former treatment clusters will receive no LLMLs. This strategy will minimize ascertainment biases that might be attributed to care-seeking behaviors of the population or to malaria detection and reporting by malaria treatment clinics. We will test LLMLs manufactured by Central Life Sciences. The larvicide application regime is as follows: temporary, semipermanent, and permanent habitats will be treated with FourStar controlled release granule formulation, 90-day briquettes, and 180-day briquettes, respectively. Application dosage will follow the recommendation of the manufacturer: 10 lbs per acre of water surface for the granule formulation, and 100 ft^2^ water surface per briquette.

We will conduct monthly vector surveys throughout the study period (Fig. 3) to determine indoor- and outdoor-biting vector abundance, using the same sample size of 64 trap-nights per cluster per month, and sporozoite infection and mosquito blood meal analysis will be conducted on all collected specimens. To confirm larviciding efficacy, we will examine larval abundance, age structure, and pupal productivity on a monthly basis in 100 randomly selected larval habitats each from treatment and control sites using our GIS maps and data on sites where LLML was applied.

#### Sample size justification

Sample size was calculated based on 2010 and 2011 active case surveillance results from Iguhu and Emutete areas [47]. Then the number of clusters required and the number of individuals required for each cluster were calculated following the methods developed by Hayes and Bennett based on cluster-randomized trials assuming equal population for each cluster [59]. The observed malaria incidence rate was 52.7 cases per 1000 people year in 2011. We calculated the numbers of clusters (matched-pairs) and individuals required for epidemiological (clinical malaria) assessment of the long-lasting larvicide treatments to detect a 50 % protective efficacy conferred by the treatment compared with the reference group (no treatment), with a power of 80 %, significance level of 5 % and the coefficient of variation of true proportions between clusters within each treatment was assumed to be 0.15. The estimated number of clusters (matched-pairs) for the intervention will be five and the required number of individuals for each matched-pair will be 1196; assuming a design effect of 0.25 and 20 % of subjects lost to follow-up. The estimated number of clusters (matched-pairs) for the intervention will be seven and the required number of individuals for each of the matched-pairs will be fewer than 2000. The 28 clusters proposed in the randomized cluster study will detect 50 % malaria incidence reduction with 99.9 % power and 30 % incidence reduction with 85.3 % power. This is based on the current malaria incidence rate in the study sites (52 clinic cases per 1000 population year) and a two-tailed alpha with a human population size of 2000 per cluster (Table 1). If the malaria incidence is 50 % lower than the current value, the design will still detect 50 % incidence reduction with 99.7 % power and 40 % reduction with 95.2 % power (Table 1).

**Table 1:** Calculated power (%) to detect various levels of incidence reduction under three incidence scenarios

Annual incidence rate (cases/1000 population)	Malaria incidence reduction			
	50 %	40 %	30 %	20 %
Observed in the site: 52	99.9	98.7	85.3	48.6
Low-incidence value: 26	99.7	95.2	74.4	38.9
High-incidence value: 78	99.9	99.3	89.0	53.0

#### Data analysis

We will monitor primary and secondary endpoint outcomes throughout the 5-year study period (Fig. 3); data analysis will be conducted in year 5. The difference in clinical malaria incidence between treatment and control groups will be compared using Poisson multivariate regression models with intervention, age, and calendar time as covariates, using a generalized estimating equations (GEE) approach. GEE is necessary since incidence will be modeled monthly as a temporally-correlated repeated measure using grouped data. Intervention will be a time-varying covariate since the treatment crosses over after three intervention rounds. Since there is no intervention in the 8 months during the washout period, interval censoring will be performed to exclude the second 4 months of data during this period. The odds ratio and the 95 % confidence interval for clinical malaria rates between treatment and control groups will be calculated. Difference in vector density and EIR will be analyzed using a negative binomial regression model and the GEE approach. In all these analyses, clusters will be indicated as intervention and control, calendar time will be categorized into: pre intervention, intervention, postintervention (4 months), washout (4 months), crossover intervention, postintervention (4 months), and nonintervention, and months since intervention (for both first intervention and crossover) will also be included as an independent variable. These variables will allow for comparison between intervention and control clusters based on baseline observations, e.g., relative reduction in vector density, and allow for evaluation of cumulative effect, e.g., the second round of treatment may produce added-effect following first-round treatment [49, 60, 61].

Finally, for the economic evaluation, we will calculate incremental cost-effectiveness ratios (ICERs) based on the primary endpoint (clinical malaria cases prevented) and on long-term health outcomes including malaria deaths averted. Using the “ingredients approach” [62, 63], costs will be classified according to: initial setup investment (e.g., capital for vehicles used in transporting larvicides, GPS units for habitat mapping, storage space and equipment, and traps for mosquito surveillance), running costs (e.g., long-lasting larvicides, salary for larvicide application staff, staff training, protective clothes, gloves, fuel costs, and vehicle insurance), and costs of program management and quality control (e.g., material procurement, project coordinator, and quality controller). Cost data will be estimated from health facility and Ministry of Health records, LLML manufacturers and financial accounts of the research project. One-way and multi-way sensitivity analysis will be undertaken to examine the implications of potential changes in variables such as larvicide price and larviciding application frequency. ICERs will be reported from both provider and societal perspectives for different transmission intensity scenarios.

## Discussion

Larval control and environmental management have played very important roles in malaria elimination in the United States and Europe, where today larval control using biological larvicides is the primary vector control method [64–66]. Larvicides target mosquito larvae, representing a major advantage over adult control, in which changes in biting and resting behaviors can lead adult mosquitoes to evade control activities. In addition, microbial larvicides from bacteria *Bti* and *Bs* have different modes of action than pyrethroid insecticides; therefore, microbial larvicides do not aggravate pyrethroid resistance. Microbial larvicides are also considered safe for non-target organisms and human health [67]. Furthermore, larval control does not conflict with but rather complements the front-line ITN and IRS malaria control programs [66]. Larval control may now be timelier than ever, since pyrethroid resistance and outdoor malaria transmission are increasing in Africa.

However, there are some potential limitations of larviciding as it is practiced today. Although there are three formulations of long-lasting larvicide available for use in different habitat types (i.e., temporary habitats, semipermanent habitats, and permanent habitats), the classification of habitats is primarily based on the longevity of the aquatic period and productivity of the habitat. The longevity of the aquatic period may be visually identified; however, the productivity of a habitat may change over time [68–71]. Canopy cover in the habitat, such as grasses in the water, may affect the spread of *Bti*/*Bs* [Zhou, personal observations]. Furthermore, heavy rainfall may wash away *Bti*/*Bs* and create new habitat; therefore, additional *Bti*/*Bs* may need to be applied at an unplanned time after the rain.

There are also limitations for the design. The incidence of clinical malaria is essential for the evaluation of intervention success. However, as pointed out by previous studies [47, 72], crude health facility records are not always a reliable source of such information and may in fact under estimate the true clinical incidence rate [47, 72]. However, as long as clinical malaria was diagnosed the same way across all health care facilities, comparison between intervention and control groups is justified. EIR is a good measure of reduction in transmission since larval control reduces overall vector population density and EIR is measured based on vector population density. Additional indicators, such as clinical incidence through active case surveillance, can be a more accurate estimate of incidence, and parasite prevalence through cross-sectional surveillance may be helpful. However, as per restrictions imposed by the funding policy, direct measures of human subjects are restricted.

Despite very high bed net coverage, malaria incidence in many African sites is resurging after a short-time reduction when ITN and IRS scale-up was initially rolled out. This malaria resurgence is caused primarily by increases in insecticide resistance and outdoor transmission. New cost-effective methods beyond bed nets and IRS are urgently needed. Long-lasting microbial larviciding represents a promising new tool that can target both indoor and outdoor transmission and alleviate the problem of pyrethroid resistance. Comprehensive evaluation of potentially cost-effective LLML will provide critically needed data for determining whether LLML can be used as a supplemental malaria control tool to further reduce malaria incidence in Africa.

### Trial status

This trial is still in its early stages. Retrospective collection of clinical malaria in the study area is underway. Larvicide importation permission has been approved from the Pest Control Products Board of Kenya. No recruiting or larvicide application has been started.

## Abbreviations

ANOVA: Analysis of variance
*Bs*: *Bacillus sphaerius*
*Bti*: *Bacillus thuringiensis israelensis*
CDC: US Centers for Disease Control and Prevention
DOMC: Division of Malaria Control
EIR: Entomological inoculation rate
ELISA: Enzyme-linked immunosorbent assay
EPA: US Environmental Protection Agency
GEE: Generalized estimating equations
GIS: Geographic information system
GPS: Global positioning system
IRS: Indoor residual spraying
ITN: Insecticide-treated nets
LLML: Long-lasting microbial larviciding
PCR: Polymerase chain reaction
PSC: Pyrethrum spray collections
